# UBXN7 facilitates SARS-CoV-2 replication via inhibiting the K48-linked ubiquitination of viral N protein

**DOI:** 10.1371/journal.ppat.1013593

**Published:** 2025-10-14

**Authors:** Tian Xia, Min Luo, Yuncheng Wang, Yaping Qin, Xiaoning Li, Shuying Chen, Junqi Xiang, Shanrong Yang, Yaokai Wang, Jing Zhu, Bo Yang, Li Lin, Jiajun Yan, Yunxiao Dou, Jian Shang, Na Zang, Yong Lin, Xiaohong Yao, Yushun Wan

**Affiliations:** 1 College of Basic Medicine, Chongqing Medical University, China; 2 Institute of Pathology, Southwest Hospital, Third Military Medical University (Army Medical University), and Key Laboratory of Tumor Immunopathology, Ministry of Education of China, Chongqing, China; 3 Henan Institute of Medical and Pharmaceutical Sciences, Zhengzhou University, Zhengzhou, China; 4 Department of Respiratory Children’s Hospital of Chongqing Medical University, National Clinical Research Center for Child Health and Disorders, Ministry of Education Key Laboratory of Child Development and Disorders, Chongqing Key Laboratory of Child Rare Diseases in Infection and Immunity, China; 5 Key Laboratory of Molecular Biology of Infectious Diseases (Chinese Ministry of Education), Chongqing Medical University, China; National Institute of Allergy and Infectious Diseases, UNITED STATES OF AMERICA

## Abstract

Host factor-mediated post-translational modification of coronavirus proteins has been demonstrated as an important strategy for regulating viral proliferation. Identification of key host genes involved in this process may provide potential therapeutic targets. In this study, we used the complementary reverse genetic system to determine that UBXN7 promotes SARS-CoV-2 viral double-stranded RNA (dsRNA) production and also promotes the replication of other human coronaviruses. However, UBXN7 does not affect the replication of VSV and RSV, suggesting that it may be a potential pan human coronaviral anti-infection target. Our results revealed that UBXN7 did not affect the viral invasion of cells, but instead hijacked viral genome assembly by interacting with SARS-CoV-2 N protein via its UBX domain. Further data indicated that UBXN7 inhibits K48-linked ubiquitination and proteasomal degradation of SARS-CoV-2 N protein, leading to N protein accumulation. Moreover, K257 of N protein was identified as specific target site of UBXN7 which are critical for viral replication. These findings reveal a novel relationship between host gene-mediated protein ubiquitylation and viral genome assembly, providing new strategies for potential pan-coronavirus drug design.

## Author summary

Coronaviruses, including SARS-CoV-2, rely on host cellular machinery to complete their life cycle. Here, we identify UBXN7, a host ubiquitin-regulatory protein, as a critical facilitator of SARS-CoV-2 and other human coronaviruses. UBXN7 promotes viral replication by directly binding to the viral nucleocapsid (N) protein through its UBX domain, thereby inhibiting K48-linked polyubiquitination of the N protein. This interaction prevents proteasomal degradation of the N protein, allowing its accumulation and enhancing its ability to bind viral genomic RNA—a key step in virion assembly. Strikingly, UBXN7 specifically targets conserved lysine residue K257 on the N protein, which are essential for viral genome assembly. Importantly, UBXN7’s proviral role is selective, as it does not influence the replication of unrelated viruses like VSV or RSV. Our findings uncover a novel mechanism by which coronaviruses hijack host ubiquitination pathways to stabilize viral proteins and drive infection. Targeting the UBXN7-N protein axis could offer a promising strategy for developing broad-spectrum antivirals against current and emerging coronaviruses.

## 1. Introduction

Severe acute respiratory syndrome coronavirus 2 (SARS-CoV-2) is a *Betacoronavirus* that caused the global pandemic, and can cause severe pneumonia or even multiple organ failure, resulting in over 7 million deaths worldwide. However, the mechanisms of the viral-host interaction, which could inform the development of novel antiviral drugs, are still not fully understood [1–3]. Although several drugs, antibodies, and vaccines have been developed against SARS-CoV-2 infection, the high mutagenicity leads to immune evasion and resistance, which is still a stumbling block in clinical practice [4,5]. However, therapies targeting host factors involved in the viral response would not be susceptible to such genetic variability [6–8]. Therefore, a deeper understanding of virus-host interactions at the cellular and molecular levels is crucial for the development of both preventive and therapeutic approaches.

The life cycle of coronaviruses encompasses several key stages: viral entry, replication, assembly of viral particles, transport, and release [9]. In the life cycle of SARS-CoV-2, its structural proteins play distinct and collaborative roles. The spike protein (S protein) primarily facilitates viral entry into host cells [10]. The membrane protein (M protein) and the envelope protein (E protein) are instrumental in mediating the assembly and budding of viral particles [11,12]. The nucleocapsid protein (N protein) is central to the core phase of viral replication. It binds to viral RNA to form ribonucleoprotein complexes (RNPs), safeguarding the stability of the genome and promoting RNA replication. Additionally, the N protein interacts with the M protein to complete the assembly of viral particles and, during the later stages of infection, suppresses the host immune response [13–15]. The multifunctionality of the N protein as a critical hub linking viral replication, structural assembly, and immune evasion, make it a pivotal target for antiviral interventions. Although the primary functions of these structural proteins have been preliminarily elucidated, the dynamic interactions, molecular crosstalk, and regulatory circuits among the three main factors of viral replication - viral RNA, viral proteins, and host proteins - remain poorly understood. In particular, many key host genes that mediate these processes are still unidentified.

A genome-wide CRISPR screen identified several host genes essential for coronavirus infection (ACE2, CTSL, SMARCA4, DYRK1A, and UBXN7, among others) [16]. Interestingly, the host gene UBXN7 was also found in another CRISPR SARS-CoV-2 infection screen [17], suggesting that it may be a key gene in the SARS-CoV-2 infection process. UBXN7 is a member of the ubiquitin regulatory X (UBX) protein family, of which UBXN1, UBXN9, and UBXN11 have been shown to inhibit the production of retroviruses and lentiviruses [18]. UBXN7 has four distinct domains and has been shown to bind to a number of E3 ubiquitin ligases through its UBA and UAS domains [19,20], AAA + ATPase p97 through its UBX domain [21,22], and neddylated CUL2 or CUL3 through its UIM domain [23,24]. It has also been found that HBV-encoded HBx promotes HBV replication by interacting with UBXN7 and promoting its ubiquitin-dependent degradation [25]. However, the role of UBXN7 in the pathogenesis of coronavirus is still unknown.

Here, we demonstrate that UBXN7 is an important host factor that promotes human coronavirus replication. Our studies suggest that UBXN7 promotes viral infection independently of cell entry, but rather promotes viral assembly by inhibiting K48-linked ubiquitination of the SARS-CoV-2 N protein. Mechanistically, UBXN7 promotes the interaction of the N protein with the SARS-CoV-2 genomic RNA, facilitating viral replication. Moreover, we identify K257 of the SARS-CoV-2 N protein as specific target sites of UBXN7 that are critical for viral genome assembly. In summary, this study shows that ubiquitination is important in coronavirus infection, host factor UBXN7 can participate in viral assembly though ubiquitination. These fundings provide novel insights into host-viral interactions and provides potential anti-viral target for pan-coronaviral infections.

## 2. Results

### 2.1 UBXN7 is an important host factor that promotes the replication of human coronaviruses

**Fig 1:**
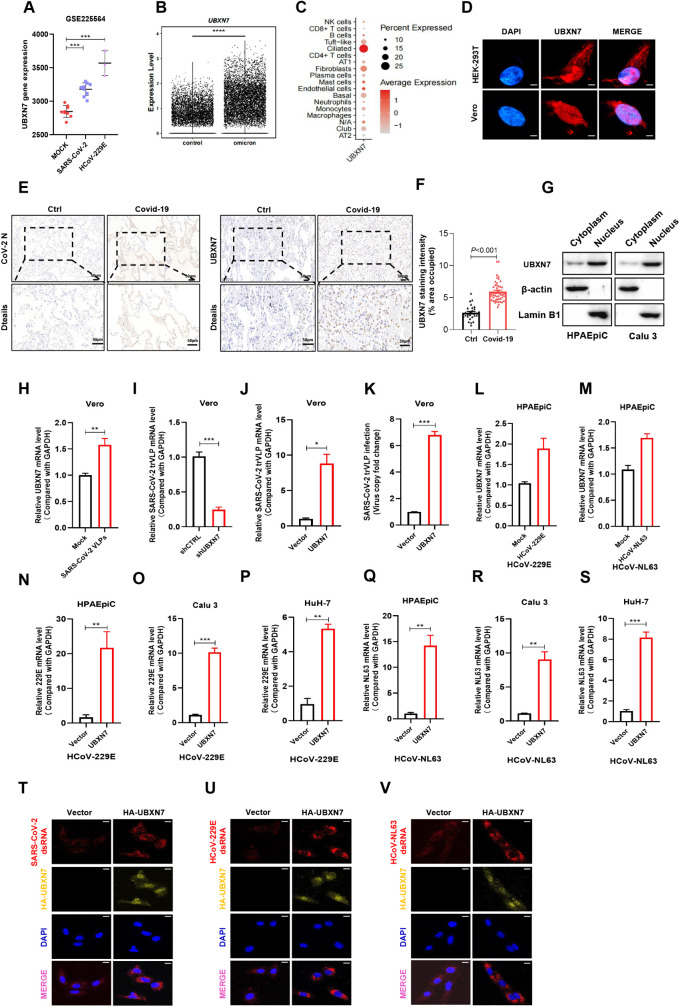
UBXN7 promotes the replication of human coronaviruses. (A) GEO data (GSE225564) analysis of UBXN7 mRNA expression in the mock (n = 9), SARS-CoV-2-infected (n = 9), and HCoV-229E-infected (n = 3) lung buds. (B) Single-cell sequencing was performed on tissue samples from healthy individuals (n = 5) and those infected with Omicron (n = 5), and the expression of UBXN7 transcripts in the control and infected groups were analyzed using the sequencing data. (C) The data map of single cell ordinal number shows the expression of UBXN7 in different cell types; the Y axis represents different cell types, the size of the point represents the percentage of cells expressing UBXN7 in each cell type, and the color of the point corresponds to the average expression level of UBXN7 in each cell type. (D) Immunofluorescence staining showed that the UBXN7 protein was mainly located in the nucleolus, with low expression in the cytoplasm. Scale bar, 50 μm. (E) IHC staining of SARS-CoV-2 N and UBXN7 expression in healthy lung tissue (n = 3) and lung tissue from COVID-19 patients (n = 10). (F) The quantitative analysis of UBXN7 expression in normal and COVID-19 lung tissues. (G) Western blot for UBXN7 expression in cytoplasmic and nuclear fractions of ESCC cells, The nuclear internal reference Lamin B and the cytoplasmic internal reference β-actin were used to detect the separation efficiency. (H) The mRNA abundance of UBXN7 in Mock and SARS-CoV-2 VLP-infected Vero cells was evaluated by qPCR. (I-J) Vero cells were infected with SARS-CoV-2 VLPs, and the changes in SARS-CoV-2 mRNA in cells after silencing or overexpressing UBXN7 were detected by qPCR. (K) qPCR detection of fold changes in viral copy number in cell supernatants. (L-M) The mRNA abundance of UBXN7 in HCoV-229E-infected and HCoV-NL63-infected HPAEpiC cells were assessed by qPCR. (N-P) qPCR was used to evaluate the effect of UBXN7 on viral genomic RNA transcription in HCoV-229E-infected HPAEpiC, Calu 3 and HuH-7 cells. (Q-S) qPCR was used to evaluate the effect of UBXN7 on viral genomic RNA transcription in HCoV-NL63-infected HPAEpiC, Calu 3 and HuH-7 cells. (T-V) HEK293 cells were transfected with HA-UBXN7 and vector plasmids for 24 h, then infected with SARS-CoV-2 VLPs, HCoV-229E, or HCoV-NL63 for 24 h. Afterward, cells were fixed with 4% paraformaldehyde, labeled with dsRNA antibody (red), HA antibody (yellow), and the nuclei counterstained with DAPI (blue). Confocal microscopy was used to analyse viral dsRNA replication in the different groups. Significant differences: * P < 0.05, ** P < 0.01, and *** P < 0.001. “ns” indicates no significant difference.

As UBXN7 has been identified in screens as a potentially important host factor for human coronavirus infection [16,17], we aimed to confirm this role. First, to confirm the association of UBXN7 with SARS-CoV-2 infection, we used the Human lung tissue with COVID19: GSE225564 data set of the public GEO (Gene Expression Omnibus) database. Our analysis showed that UBXN7 gene expression was significantly increased in samples infected with SARS-CoV-2 and HCoV-229E compared with the uninfected group (Fig 1A). Moreover, single-cell sequencing data analysis revealed that UBXN7 gene expression was higher in tissues infected with SARS-CoV-2 (Omicron) compared to normal lung tissues (Fig 1B). The upregulation of UBXN7 in SARS-CoV-2-infected tissues suggests that UBXN7 may play a regulatory role in viral infection. Interestingly, the results of single cell sequencing data show that Omicron infection promotes the high expression of UBXN7 predominantly in ciliated cells, one of the most susceptible cell-types in the upper respiratory tract, and target cells for viral replication. Therefore, we hypothesize that the up-regulation of UBXN7 may promote viral replication (Fig 1C). Immunofluorescence results showed that UBXN7 was expressed in both the nucleus and cytoplasm (Fig 1D). In addition, we analyzed the expression of UBXN7 in 10 lung tissue from COVID-19 patients and 3 lung tissues from healthy individuals by immunohistochemical staining with UBXN7 antibody. We first confirmed the presence of the SARS-CoV-2 virus in the collected COVID-19 lung tissues by immunohistochemical staining for the CoV-2 N protein (Fig 1E). Subsequently, we examined the expression levels of UBXN7 in both COVID-19 and normal lung tissues. The quantitative results showed that the expression of UBXN7 in COVID-19 lung tissue was higher than that in normal lung tissues, and the staining site was in both the nucleus and cytoplasm (Fig 1E-1F). The nuclear-cytoplasmic separation results showed that UBXN7 was mainly located in the nucleus, with low expression in the cytoplasm (Fig 1G). To study the role of UBXN7 in SARS-CoV-2 infection, we used a safe virus-like particle (trVLP) system that can be used in BSL-2 laboratories [26,27]. We constructed trVLP lacking the N gene but carrying a GFP reporter gene (SARS-CoV-2 GFP/ΔN) (S1A Fig). The trVLP genome was assembled by PCR amplification of fragments containing the 5’ and 3’ UTRs, an N-deficient SARS-CoV-2 genome, and GFP, followed by gel purification (S1B-S1G Fig). Full-length genomic RNA (FL-RNA) and separate N gene mRNA were generated via in vitro transcription (. S1H-S1J Fig), ensuring that trVLP could only replicate in cells trans-complemented with N protein. To validate the trVLP system, we infected Vero-N cells with SARS-CoV-2 GFP/ΔN trVLP. Fluorescence microscopy confirmed GFP expression, indicating successful viral entry and replication (S1K Fig). This system can recapitulate the complete viral life cycle in cells expressing the SARS-CoV-2 N protein which is widely used for coronavirus studies. Vero-N cells infected with SARS-CoV-2 trVLPs displayed increased levels of UBXN7 mRNA (Fig 1H), consistent with our previous data. Subsequently, the potential role of UBXN7 in SARS-CoV-2 infection was analyzed through gain- or loss-of-function. First, we evaluated the knockdown efficiency of UBXN7 in Vero cells and selected a shUBXN7 sequence with optimal efficacy (S1L-S1M Fig). RT-qPCR detection of intracellular viral RNA levels showed that knocking down UBXN7 inhibited the copying of viral genomic RNA (Fig 1I), while overexpression of UBXN7 promoted SARS-CoV-2 genome copying (Fig 1J). The viral load of SARS-CoV-2 in the supernatant of overexpressed UBXN7 cells also increased by nearly 7 times (Fig 1K). The above results indicate that UBXN7 is an important factor promoting SARS-CoV-2 infection.

We next infected respiratory epithelial cells (HPAEpiC and Calu 3) and hepatocellular carcinoma cells (Huh-7) with HCoV-229E and HCoV-NL63, two other pneumonia-inducing human coronaviruses, to further assess the role of UBXN7 in coronavirus infection. HPAEpiC cells infected with two human coronaviruses displayed increased levels of UBXN7 mRNA (Fig 1L and 1M). In addition, RT-qPCR revealed that the levels of HCoV-229E or HCoV-NL63 cytoplasmic RNAs were significantly increased by overexpression of UBXN7 in each cell types (Fig 1N-1S), representatively. Immunofluorescence staining of viral dsRNA showed increased signal intensity in UBXN7-overexpressing Vero cells infected with SARS-CoV-2, HCoV-229E, or HCoV-NL63 compared to control cells, suggesting that UBXN7 expression enhances coronavirus replication. (Fig 1T-1V). Next, we examined the effect of UBXN7 on vesicular stomatitis virus (VSV) and respiratory syncytial virus (RSV) to evaluate whether UBXN7 has similar effects on other virus types. We infected cells with VSV expressing green fluorescent protein (VSV-GFP) and RSV expressing red fluorescent protein (RSV-mCherry) and quantified the number of infected cells by microscopy. The results showed that UBXN7 did not affect the infection efficiency of VSV and RSV (S2A-S2D Fig). This suggests that the proviral effect of UBXN7 may be specific to coronaviruses. Collectively, these data indicate that UBXN7 is an important host factor that promotes the replication of human coronaviruses.

### 2.2 UBXN7 does not affect the cell entry process of SARS-CoV-2

**Fig 2:**
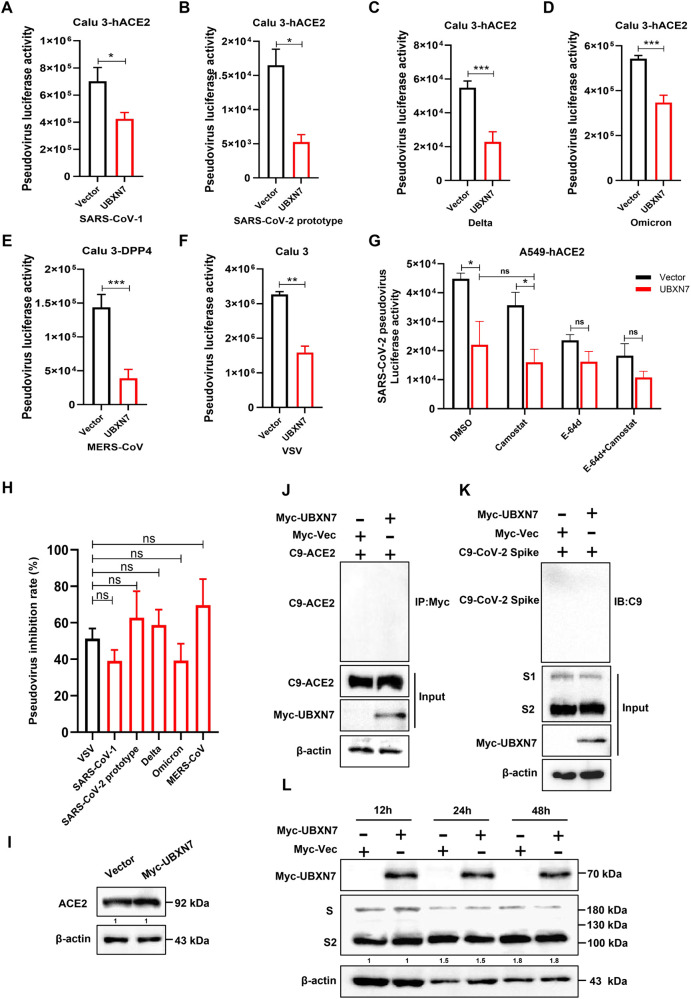
UBXN7 does not affect the invasion of human coronavirus pseudoviruses. (A-F) Calu-3 cells were infected with SARS-CoV-1 pseudovirus, SARS-CoV-2 prototype pseudovirus, Delta strain pseudovirus, Omicron strain pseudovirus, MERS-CoV pseudovirus, or VSV pseudovirus. Luciferase values were measured to evaluate the pseudovirus invasion efficiency of cells in the vector group and UBXN7 overexpression group. (G) Quantification of SARS-CoV-2 pseudovirus infection in A549 cells of the control or UBXN7 overexpression groups treated with the indicated compounds (E-64d: 20 μM, Camostat: 20 μM). (H) Comparison of pseudovirus inhibition rates between different experimental groups and the VSV control group. “ns” indicates no significant difference. (J and K) Co-IP analysis of the interaction between UBXN7 and ACE2 or SARS-CoV-2 Spike protein in HEK293T cells. (I) Western blot analysis of UBXN7 affecting ACE2 expression levels in cells. (L) Cleavage of the SARS-CoV-2 spike protein in Vector or UBXN7 overexpressing HEK293T-ACE2 cells infected with pseudovirus carrying SARS-CoV-2 spike protein for the indicated time periods. Significant differences: * P < 0.05, ** P < 0.01, and *** P < 0.001. “ns” indicates no significant difference.

Next, we sought to determine which step of the viral life cycle is targeted by UBXN7. First, to evaluate the viral entry process, we constructed a pseudovirus assay using a replication-defective HIV-1 vector as a backbone and the coronavirus spike protein as an envelope protein [28]. Calu3 cells with ACE2 receptors were infected with pseudoviruses of SARS-CoV-1, SARS-CoV-2 (ancestral and variants), while Calu3 cells with DPP4 receptors were infected with pseudoviruses of MERS-CoV, and VSV pseudovirus was used as a negative control. The results showed that overexpression of UBXN7 inhibited the infectivity of all pseudoviruses with spike proteins, including the control VSV pseudovirus (Fig 2A-2F). Furthermore, statistical analysis revealed that there were no significant differences in the pseudovirus inhibition rates between all experimental groups and the VSV group (Fig 2H). This suggests that UBXN7 inhibited the HIV virus backbone rather than the cell entry process of coronavirus.

In order to verify the reliability of this conclusion, we further dissected the influence of UBXN7 on the specific steps of coronavirus invasion. SARS-CoV-2 uses two fusion pathways to invade host cells: the cathepsin-dependent endosome fusion pathway and the TMPRSS2-dependent cell surface fusion pathway. The cells infected by SARS-CoV-2 pseudovirus were therefore treated with a saturated concentration of E-64d (a cathepsin L inhibitor) or a TMPRSS2 inhibitor to separate these two pathways. We found that UBXN7 had no significant effect on either of the two methods of virus invasion compared with the control group (Fig 2G). This demonstrated that UBXN7 did not affect the fusion and internalization of the virus membrane surface. Assays for receptor binding, adhesive internalization, and spike cleavage were also performed. Western blot and Co-IP results showed that UBXN7 did not affect the expression of ACE2 (Fig 2I), did not interact with ACE2 or SARS-CoV-2 S protein (Fig 2J and 2K), and did not affect the cleavage of S protein (Fig 2L). Therefore, we conclude that UBXN7 does not affect the invasion process in the SARS-CoV-2 life cycle.

### 2.3 Interaction between UBXN7 and SARS-CoV-2 N protein

**Fig 3:**
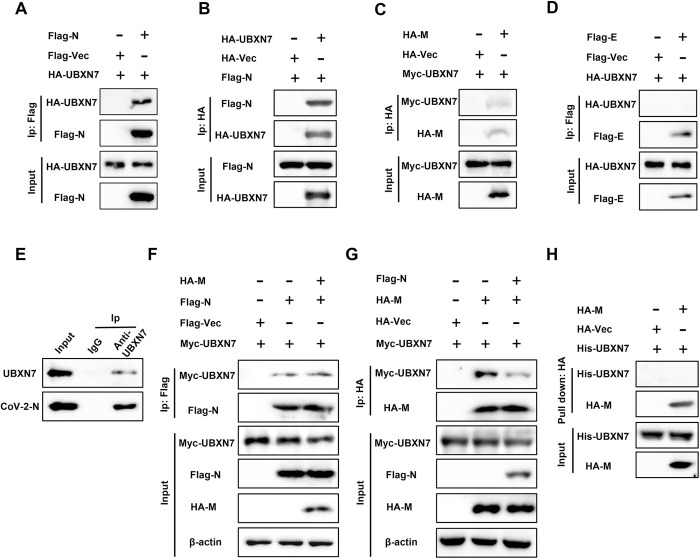
UBXN7 interacts with SARS-CoV-2 N protein. (A-D) Co-IP analysis of the interaction between the viral structural protein and UBXN7. HEK293T cells were cotransfected with UBXN7 and SARS-CoV-2 protein plasmid constructs. Total cell lysates and immunoprecipitates were analyzed by western blotting after immunoprecipitation with FLAG or HA antibodies. Immunoprecipitated samples were probed with anti-HA or anti-Myc antibodies. (E) Endogenous interaction between UBXN7 and N protein in HPAEpiC cells. Co-IP was performed using anti-UBXN7 antibody or control IgG in HPAEpiC cell lysates, followed by immunoblotting with antibodies against N protein and UBXN7. (F) Co-IP analysis to determine whether the interaction between UBXN7 and the N protein is disrupted by the M protein. HEK293T cells were co-transfected with UBXN7, M, and N plasmids for 48 h, followed by cell lysis and immunoprecipitation using an anti-Flag antibody. Immunoblotting was then performed using an anti-Myc antibody. (G) Co-IP analysis to determine whether the interaction between UBXN7 and the M protein is disrupted by the N protein. HEK293T cells were co-transfected with UBXN7, M, and N plasmids for 48 h, followed by cell lysis and immunoprecipitation using an anti-HA antibody. Immunoblotting was then performed using an anti-Myc antibody. (H) Purified recombinant UBXN7-his protein was incubated with cell lysate containing M-HA protein overnight at 4°C. Subsequently, M protein complexes were enriched using HA-magnetic beads, and UBXN7 protein was detected by immunoblotting using anti-his antibody.

To clarify the role of UBXN7 in viral replication and assembly, we cloned structural protein genes of SARS-CoV-2 into mammalian expression plasmids and tagged them with FLAG or HA tags to facilitate the detection of their expression. The viral protein plasmids and UBXN7 plasmids were co-transfected into HEK293T cells for 48 hours, and the cell lysates were subjected to co-immunoprecipitation experiments. This revealed a strong interaction between UBXN7 and N proteins, and a much weaker interaction with M proteins (Fig 3A-3D). Endogenous Co-IP in HPAEpiC cells demonstrated interaction between UBXN7 and N protein (Fig 3E). The N and M proteins are involved in the assembly of virion production. Competitive IP binding experiments demonstrated that UBXN7 is capable of independently binding to the N protein, and this interaction is not influenced by the M protein (Fig 3F). However, when UBXN7 binds to the M protein, the N protein competes with the M protein, thereby interfering with the UBXN7-M protein interaction and pull-down assay results indicate that UBXN7 does not directly interact with the M protein, their binding may be indirect (Fig 3G and 3H). Moreover, Co-IP assays in HEK293T cells demonstrated that HA-UBXN7 interacts with both HCoV-229E N and HCoV-NL63 N proteins (S3A and S3B Fig). This suggests that UBXN7 may primarily exert its proviral effects through the N protein.

### 2.4 SARS-CoV-2 N protein interacts with the UBX ubiquitin domain of UBXN7

**Fig 4:**
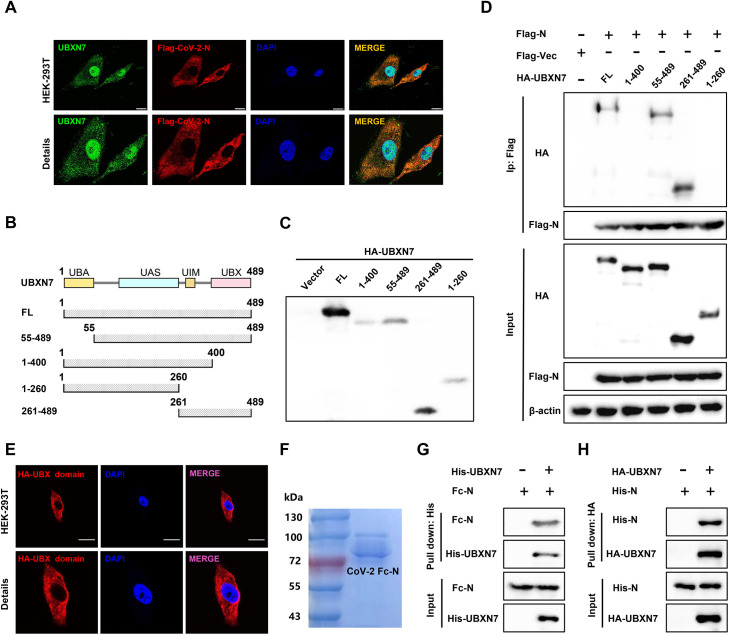
The UBX domain of UBXN7 interacts with N protein. (A) HEK293 cells transfected with the indicated plasmid were analyzed by confocal microscope. N protein and UBXN7 were labeled with anti-Flag antibody (red) and UBXN7 antibody (green) respectively. The nucleus was stained with DAPI (blue). Display representative images. Scale, 10 μm. (B) The diagrams of UBXN7 structural domains are presented. (C) The HA-tagged UBXN7 truncated plasmid was transfected into HEK293T cells, and protein expression was detected by Western blotting. (D) Plasmids expressing Flag-tagged N and HA-tagged UBXN7 (1–489aa), UBXN7 (1–400aa), UBXN7 (55–489 aa), UBXN7 (261–489aa), and UBXN7 (1–260aa) were co-transfected into HEK293 cells, and the input and immunoprecipitated proteins were detected using anti-Flag and anti-HA antibodies and analyzed by Western blotting for interacting domains. (E) Subcellular localization of the HA-tagged UBX domain of UBXN7 in transfected HEK293T cells. HEK293T cells were transiently transfected with a plasmid encoding the HA-tagged UBX domain of UBXN7. Immunofluorescence staining was performed using anti-HA antibody (red) and DAPI (blue) to label nuclei. Scale, 10 μm. (F) Fc-tagged N protein was expressed in eukaryotic cells, purified by affinity chromatography and detected by Coomassie Brilliant Blue staining. (G) Purified recombinant UBXN7-his protein was incubated with N-Fc protein overnight at 4°C. Subsequently, N protein complexes were enriched using his-magnetic beads, and N protein was detected by immunoblotting using anti-Fc antibody. (H) Purified recombinant His-N protein from E. coli (to avoid eukaryotic post-translational modifications) was immobilized on Ni-NTA beads and incubated with purified HA-UBXN7. Bound proteins were eluted and analyzed by Western blotting using anti-HA and anti-His antibodies.

Next, we explored which structural domain of UBXN7 interacts with SARS-CoV-2 N protein. Immunofluorescence results showed that UBXN7 and N protein partially co-localized in the cytoplasm (Fig 4A). Next, we constructed truncation and deletion mutants of UBXN7 to explore the key domains of N protein binding to UBXN7 (Fig 4B and 4C). Co-IP experiments showed that the UBX domain of UBXN7 was capable of independent interaction with N protein, and UBXN7 lacking the UBX domain could not interact with SARS-CoV-2 N protein (Fig 4D). Immunofluorescence analysis revealed that no significant nuclear accumulation of the HA-tagged UBX domain was observed, indicating its specific cytoplasmic localization (Fig 4E). In order to clarify whether the interaction between UBXN7 and N protein is direct or indirect, we purified UBXN7 and N proteins by affinity chromatography for pulldown experiments (Figs 4F and S3C). The results showed that UBXN7 and N protein interacted directly (Fig 4G). The prokaryotic-purified His-N protein directly bound HA-UBXN7 in pull-down assays, demonstrating their intrinsic physical interaction independent of eukaryotic post-translational modifications (Fig 4H). These results together confirmed that UBXN7 closely interacts with the N protein, and the interacting domain is the UBX domain, suggesting that UBXN7 may promote viral replication predominantly by regulating the N protein.

### 2.5 UBXN7 inhibits K48-linked ubiquitination and proteasomal degradation of SARS-CoV-2 N protein

**Fig 5:**
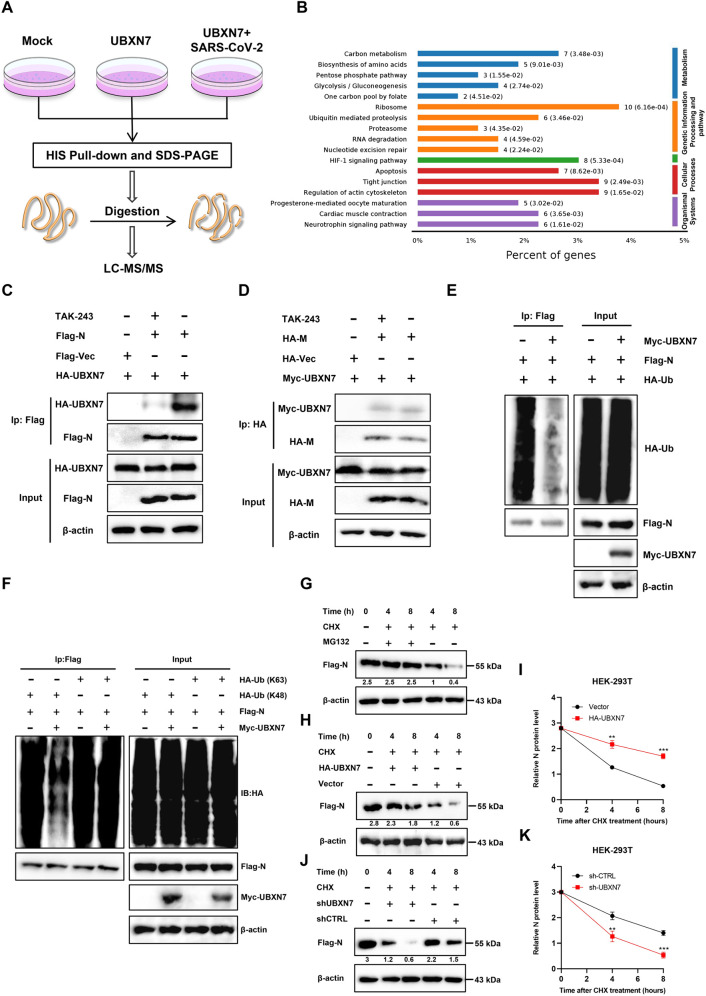
UBXN7 inhibits K48-linked polyubiquitination of the SARS-CoV-2 N protein and proteasomal degradation. (A)The experimental flow chart of analyzing the potential protein regulated by UBXN7 in SARS-CoV-2 infection state by His Pull-down and LC-MS/MS. (B) KEGG pathway analysis of LC-MS data. (C-D) After using the ubiquitin inhibitor (TAK-243, 100 nM), Co-IP assays were performed to detect changes in the interaction between UBXN7 and N or M proteins. (E) HEK293 cells were co-transfected with Myc-UBXN7, Flag-N and HA-Ub ubiquitin plasmids, and then immunoprecipitated with tag antibodies, followed by immunoblotting to detect ubiquitin levels. (F) Co-transfect the Flag-tagged N plasmid and HA-tagged K48-Ub or K63-Ub plasmid into HEK293 cells. Based on experimental groups, co-transfect the UBXN7 plasmid and confirm the ubiquitin chain on the N protein. (G) Transfect the N plasmid into HEK293 cells. 24 hours post-transfection, treat cells with cycloheximide (CHX, 10 μM) and MG132 (5 μM) according to the time gradients. Use Western blotting to detect N protein expression levels. (H-K) Western blot analysis of N protein levels in UBXN7-knockdown (shUBXN7), UBXN7-overexpressing (HA-UBXN7), and vector control cells following CHX treatment for 0, 4, and 8 h (H, I), and the line graph of UBXN7 expression (J, K) was further quantitatively analyzed. ImageJ was used to normalize actin for protein quantification, image J was used to normalize actin for protein quantification.

To further elucidate the potential mechanism by which UBXN7 promotes the viral replication process, we performed pulldown and MS to identify potential proteins regulated by UBXN7 under SARS-CoV-2 infection (Fig 5A). KEGG and GO enrichment analysis showed that the main function of UBXN7 may be related to the regulatory protein ubiquitin pathway (Figs 5B and S3D). To investigate whether UBXN7 mediates the ubiquitination of SARS-CoV-2 N protein, we performed Co-IP with ubiquitin activating enzyme inhibitor TAK-243. This revealed that the interaction between UBXN7 and N protein was inhibited by TAK-243, indicating that UBXN7 may recognize the ubiquitin chain of N protein (Fig 5C). The SARS-CoV-2 M protein was used as a control, and we detected no significant change in the binding level of UBXN7 to M protein under TAK-243 treatment (Fig 5D), suggesting that UBXN7 may exert its effects by regulating the ubiquitin/deubiquitin pathway of N protein. Ubiquitin-based immunoprecipitation analysis showed that polyubiquitination of N protein was inhibited in the presence of UBXN7 (Fig 5E), suggesting that UBXN7 inhibits N protein ubiquitination.

Given the above results, we sought to determine the type of ubiquitin chain that UBXN7 modifies on the N protein and to resolve its functional effects. The two most common ubiquitin modes in N protein are K48- and K63-linked polyubiquitination. Therefore, we performed ubiquitination assays of N protein with K48 and K63 mutants to separate the two pathways. We found that UBXN7 inhibited K48-linked polyubiquitination of N protein (Fig 5F), suggesting that UBXN7 inhibits the formation of K48-branched polyubiquitin chains on N protein. Given that K48 ubiquitin chains are usually associated with protein stability and proteasome degradation pathways, we treated HEK293 cells transfected with N protein plasmids with cycloheximide (CHX) to inhibit protein synthesis and MG132 to inhibit protease activity. There was no significant change in the expression level of N protein in the MG132-treated group. In contrast, the expression level of N protein in the MG132-untreated group was significantly downregulated (Fig 5G). These results indicate that the degradation of N protein is critically dependent on the proteasome, consistent with our previous experiments. To verify the role of UBXN7 in regulating N protein, cycloheximide (CHX), an inhibitor of protein synthesis, was used to evaluate protein stability. The data showed that overexpressing UBXN7 significantly increased the half-life of N protein and inhibited its degradation of N protein in HEK293 cells (Fig 5H and 5I). On the contrary, UBXN7 deletion significantly reduced the half-life of N protein in HEK293 cells, revealing that knockdown of UBXN7 promoted the degradation of N protein (Fig 5J and 5K). These findings suggest that UBXN7 may promote viral replication by inhibiting K48-linked ubiquitination and proteasome degradation of N protein, leading to the accumulation of N protein.

### 2.6 UBXN7 targets K257 of the SARS-CoV-2 N protein

**Fig 6:**
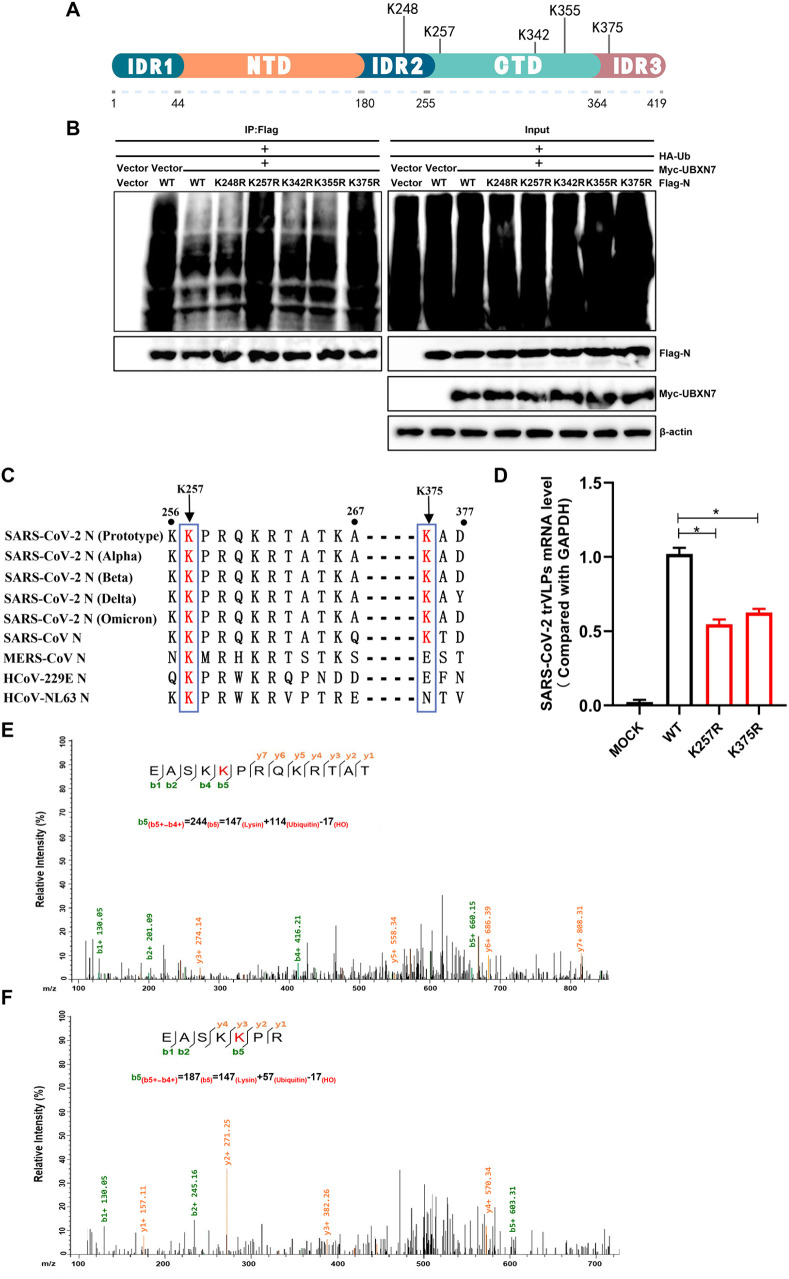
K257 of the SARS-CoV-2 N protein are specific targeting site for UBXN7. (A)Schematic diagram depicting the potential ubiquitination sites on the SARS-CoV-2 N protein. (B) Identification of specific lysine residues in the N protein targeted for modification by UBXN7 through co-transfection of Myc-UBXN7, HA-Ub, and various NP mutants with single lysine mutations. (C) Sequence alignment of the N proteins from SARS-CoV-2, SARS-CoV, MERS-CoV, HCoV-229E, and HCoV-NL63 coronaviruses. (D) Overexpress the N protein and its mutants in Vero cells, then infect them with trVLP for 48 hours. Extract RNA and use qPCR to measure the mRNA abundance of SARS-CoV-2 transcripts, evaluating the impact of N protein mutation sites on SARS-CoV-2 replication. (E) Analysis of the major product ion mass-to-charge ratios (m/z) in the MS spectra revealed that the b5 ion consists of a dehydroxylysine residue and a ubiquitin moiety, along with adjacent residues, confirming ubiquitination at Lys 257 of the N protein. (F) The decreased m/z of the ubiquitinated species upon UBXN7 overexpression demonstrated that UBXN7 suppresses ubiquitination at Lys 257. The sum of the mass-to-charge ratios (m/z) of the five amino acids (E, A, S, K, K) in the b5 + ion represents the total mass-to-charge ratio of the peptide fragment.

Based on a study that identified a series of potential ubiquitination sites on the SARS-CoV-2 N protein [29]. we selected five sites with high ubiquitin abundance (K248, K257, K342, K355, K375) for further study (Fig 6A). Point mutation screening showed that UBXN7 specifically modified the ubiquitin lysine chains at sites 257 and 375 of the N protein (Fig 6B). It is notable that K257 is highly conserved in the N protein of several coronaviruses (SARS-CoV, SARS-CoV-2, SARS-CoV-2 variants, MERS-CoV, HCoV-229E, and HCoV-NL63), and K375 exhibits relative conservation, being present only in SARS-CoV, SARS-CoV-2 and its variants (Fig 6C). To verify the role of N protein ubiquitination in SARS-CoV-2 infection, we used SARS-CoV-2 trVLPs to infect cells and detected the abundance of viral RNA in cells to assess infection efficiency. The results showed that lysine to arginine mutations at positions 257 and 375 of the N protein significantly inhibited the infectivity of SARS-CoV-2 trVLPs (Fig 6D), indicating that these two ubiquitination sites of the N protein are essential for viral survival. To further determine whether UBXN7 regulates ubiquitination at the conserved K257, we performed LC-MS/MS analysis on immunoprecipitated N protein from both the empty vector control group and the UBXN7-overexpressing group. UBXN7 significantly reduced ubiquitination of the b5 ion (m/z decreased from 244 to 187). In contrast, the unmodified b1 ion (m/z 130.05) remained unchanged under various conditions (Fig 6E and 6F), confirming the specificity of UBXN7 for K257.

### 2.7 UBXN7 promotes the binding of N protein to genomic RNA through ubiquitination

**Fig 7:**
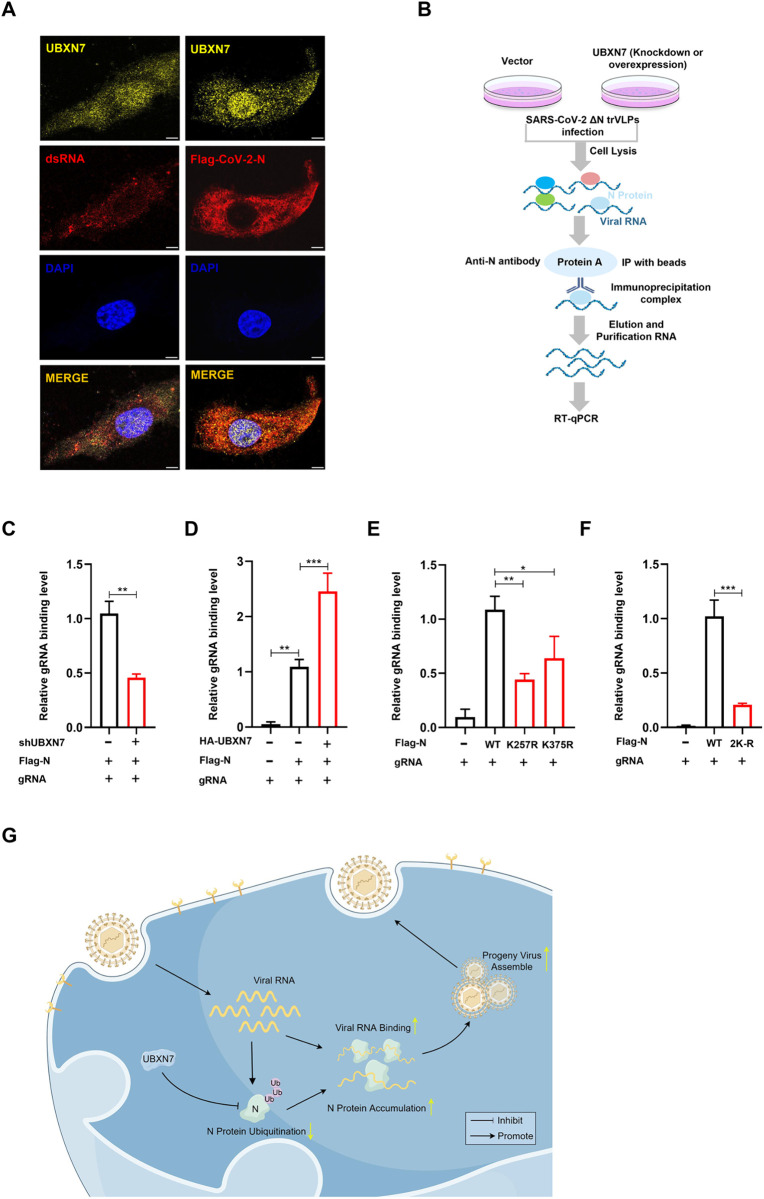
UBXN7 promotes the binding of the N protein to genomic RNA. (A) Spatial localization of UBXN7 with dsRNA and SARS-CoV-2 N protein analyzed by confocal microscopy. UBXN7 proteins (yellow), dsRNA and Flag-N (red), while the cell nucleus is stained with DAPI (blue). Overlapping localization is shown (orange). Scale bar, 10 μm. (B) Flowchart of RNA immunoprecipitation (RIP) analysis for the interaction between N protein and its genomic RNA. (C) RIP assay analysis of the endogenous UBXN7-mediated binding of N protein to gRNA in UBXN7 knockdown HEK293 cellsIn HEK293T cells with UBXN7 knockdown, RIP assay was performed to analyze the binding of N protein to SARS-CoV-2 gRNA mediated by endogenous UBXN7. SARS-CoV-2 ΔN trVLPs (MOI = 1) were used to infect both normal and UBXN7-knockdown HEK293T-N cells. The N protein was immunoprecipitated using antibodies and agarose beads to form an agarose bead-N-gRNA immunoprecipitation complex. RNA was eluted and purified from the immunoprecipitation complex, and the abundance of gRNA was detected by qPCR using specific primers to evaluate the impact of UBXN7 knockdown on the binding ability of the N protein to gRNA. (D) RIP assay analysis of changes in N protein binding to gRNA after UBXN7 overexpression. (E-F) RIP assay to detect the binding of N protein mutants to gRNA. HEK293T cells were transfected with the indicated plasmids for 24 hours, and gRNA levels were measured in different groups. (G) The mechanism map shows that UBXN7 promotes SARS-CoV-2 proliferation by inhibiting the ubiquitination of N protein to enhance the binding ability of N protein to the viral genome. The significant differences are * P < 0.05, ** P < 0.01 and *** P < 0.001. In addition, “ns” means no significance.

Studies have shown that the viral structural N protein promotes viral replication by intertwining with the viral genomic RNA. To confirm whether UBXN7 promotes viral assembly by affecting the binding of N protein to genomic RNA, we performed colocalization experiments. Confocal microscopy revealed that there was partial spatial colocalization between UBXN7, SARS-CoV-2 N, protein and viral dsRNA (Fig 7A). RNA immunoprecipitation (RIP) further evaluated the binding ability of viral N protein to genomic RNA (Fig 7B). The results showed that when UBXN7 was knocked down, the interaction between N protein and genomic RNA was significantly reduced (Fig 7C), while overexpression of UBXN7 promoted the binding of N protein to genomic RNA (Fig 7D). These results indicate that UBXN7 plays a crucial regulatory role in the binding of N protein to genomic RNA. Further experiments revealed that lysine to arginine mutations at positions 257 and 375 on N protein, both individually and in combination, significantly reduced its ability to bind to genomic RNA (Fig 7E and 7F). These results suggest that UBXN7 promotes the binding of N protein to genomic RNA through ubiquitination. Finally, we investigated whether UBXN7 indirectly affects viral replication through IFN-α and IFN-β, and the results indicated that UBXN7 does not affect the expression levels of IFN-α and IFN-β (S4A-S4J Fig).

In conclusion, these results suggest that UBXN7 inhibits K48-linked ubiquitination of N protein, leading to N protein accumulation, and that ubiquitination on 257K and 357K of N protein are critical for its ability to bind to the viral genome, promoting SARS-CoV-2 virion assembly (Fig 7G).

## 3. Discussion

Several genome-wide CRISPR/Cas9 screens revealed host factors that regulate SARS-CoV-2 infection, including some that have been shown to play important roles in SARS-CoV-2 infection [30–34]. However, most CRISPR/Cas9 screening was performed in cells overexpressing ACE2 (A549-ACE2 and Huh7.5-ACE2). This screening strategy promotes SARS-CoV-2 infection and is more likely to enrich for host factors that affect the viral invasion process by regulating ACE2 (such as SMARCA4, PLSCR1, KDM6A, DYRK1A), as confirmed in recent studies [33,35–37]. Further, this screening model may conceal many factors related to the viral replication process and some important epigenetic modification regulators. Interestingly, recent screening in Vero-E6 and Calu-3 wild-type cells identified some host factors that affect coronavirus infection independently of the ACE2 pathway, including UBXN7 [16,17].

UBXN7 is a member of the ubiquitin regulatory X (UBX) protein family, and previous studies have shown that HBV-encoded HBx protein promotes ubiquitin-dependent degradation of UBXN7 and promotes HBV replication [25]. However, it is not clear whether UBXN7 mediates the replication of coronaviruses. In this study, we proposed for the first time that UBXN7 promotes SARS-CoV-2 replication and is required for infection with multiple coronaviruses, while not affecting VSV and RSV replication. Concurrently, our virus invasion experiments revealed that UBXN7 does not affect the invasion process in the SARS-CoV-2 life cycle, and may instead play a role through the replication process. In addition, Co-IP and pulldown experiments showed that UBXN7 mainly interacts with the SARS-CoV-2 N protein to promote viral replication, which is the first report of its kind.

Previous studies have shown that N protein undergoes post-translational modification [38]. Among them, methylation, phosphorylation, and ubiquitination play crucial roles in regulating RNA binding and changing the physicochemical properties of N protein. It has been reported that arginine methylation at R95 and R177 of N protein can promote its binding to the 5’-UTR of RNA [39]. The serine-rich region of the N protein is rapidly phosphorylated at multiple sites by intracellular kinases, promoting structural changes in RNA that are required for RNA transcription [40,41]. Ubiquitination of N protein may promote the binding of its N-terminal domain to RNA, assist the assembly of the viral RNA genome into the ribonucleoprotein complex, and promote viral replication *in vivo* by interacting with M protein [42]. This all suggests that the level of N protein binding to viral RNA may depend on the balance of methylation, phosphorylation, and ubiquitination at specific sites on the N protein. Therefore, studying these modifications is important for elucidating the replication mechanism of SARS-CoV-2 and developing antiviral drugs. Considering the ubiquitination/deubiquitination function of UBXN7 [19–21,43], we confirmed that UBX, the ubiquitin structural domain of UBXN7, interacts with N protein and inhibits N protein ubiquitination. Further studies showed that UBXN7 inhibited K48-linked ubiquitination and proteasome degradation of SARS-CoV-2 N protein, and identified K257 and K375 as specific novel target sites of UBXN7. Therefore, it is reasonable to speculate that UBXN7 promotes viral replication by inhibiting ubiquitination leading to N protein accumulation. However, further studies are required to assess whether there is a link between post-translational modification of N proteins and the production of new virus particles.

Ubiquitination-mediated stabilization of the N protein by UBXN7 directly enhances viral genome packaging and assembly. Coronaviruses replicate their RNA genomes within specialized replication-transcription complexes (RTCs) in the host cytoplasm, where the N protein plays a dual role: it oligomerizes to encapsidate the genomic RNA (gRNA) into ribonucleoprotein (RNP) complexes and facilitates the recruitment of gRNA to the sites of virion assembly at ER-Golgi intermediate compartments (ERGIC) via interactions with the M protein [9,44,45]. Our data reveal that UBXN7 binds the N protein via its UBX domain, inhibiting K48-linked polyubiquitination at residue K257. This blockade of ubiquitin-dependent proteasomal degradation significantly prolongs the half-life of the N protein, leading to its accumulation. Critically, stabilized N protein exhibits enhanced binding affinity for SARS-CoV-2 gRNA, promoting the formation of stable RNPs. This is not merely a passive stabilization event; ubiquitination at K257 is essential for the N protein’s functional conformation during RNA binding, as mutations at these sites abolish gRNA interaction and cripple viral replication. Thus, UBXN7 hijacks the host ubiquitin-proteasome system to fortify the N protein pool, ensuring efficient gRNA encapsidation and subsequent RNP-N protein interactions at assembly sites. This represents a novel viral strategy to co-opt host ubiquitin regulators UBXN7 to optimize a core structural N protein for maximal virion production.

Another important function of N proteins is allowing efficient replication and transcription of the viral genome. This includes their role in the process of discontinuous transcription, which is unique to coronaviruses and occurs through the formation of sub-genomic RNAs (sgRNAs) [46]. The replication-transcription complex promotes the synthesis of dsRNA, which serves as a template for replication and transcription. The synthesized sgRNA and viral gRNA enter the cytoplasm for subsequent virion assembly and viral protein biosynthesis [44,47]. However, little is known about how viral RNAs, viral proteins, and host proteins interact to affect coronavirus replication. Our study revealed that UBXN7 facilitates the accumulation of the N protein by inhibiting its K48-linked ubiquitination, thereby enhancing the binding of N protein to SARS-CoV-2 genomic RNA and promoting viral replication. Taken together, our work identifies a novel host factor required for coronavirus replication and mechanistically links host UBXN7-mediated ubiquitination modification of the SARS-CoV-2 N protein and viral genome replication. Our results suggest that targeting the UBXN7-nucleocapsid protein axis could be a promising strategy for the development of broad-spectrum antiviral therapies for coronaviruses.

## 4. Materials and methods

### 4.1 Ethics statement

All procedures of this study involving humans (individuals, medical records, human samples, and clinical isolates) were reviewed and approved by the Ethics Committee of Southwest Hospital at Third Military Medical University, A written approval file (No. KY2020298) was obtained. All methods were performed in accordance with the relevant guidelines and regulations and adhered to the Declaration of Helsinki.

### 4.2 Viruses and cell lines

The HCoV-229E (ATCC VR-740 strain) and HCoV-NL63 (NR-470 strain) were provided by Professor Jincun Zhao (Institute of Infectious Disease, Guangzhou Eighth People’s Hospital of Guangzhou Medical University, Guangzhou 510060, China). VSV-GFP was a kind gift from Prof. Ying Zhu at Wuhan University. The RSV A2 strain were stored in our laboratory. The primary materials for constructing SARS-CoV-2 GFP/∆N virus-like particles (trVLP) were kind gifts from Professor Ding Qiang at Tsinghua University. The construction process was carried out in accordance with previously published research protocols [26,27]. All our operations with viral infection are conducted in BSL-2 laboratory.

HEK293T, Vero, HPAEpiC, Calu 3, Huh-7, and A549 cells were cultured and maintained in DMEM (Gibco) containing a penicillin-streptomycin mixture and 10% FBS, with 5% CO2 at 37 °C. All cell lines are derived from preservation in this laboratory.

### 4.3 Chemical reagents, antibodies and plasmids

Camostat (HY-165529), E-64d (HY-100229), TAK-243 (HY-100487), Cycloheximide (HY-12320) and MG132 (HY-13259) were purchased from MedChemExpress. The main antibodies were as follows: anti-UBXN7 mAb (ab185085, Abcam), anti-dsRNA mAb J2 (10010200, Scicons), mouse anti HA-Tag mAb (AE008, ABclonal), HA-Tag rabbit mAb (AE105, ABclonal), Flag-Tag rabbit mAb (AE092, ABclonal), Myc-Tag rabbit mAb (AE070, ABclonal), mouse anti Myc-Tag mAb (AE010, ABclonal), β-Actin rabbit mAb (AC026, ABclonal), HRP-conjugated goat anti-rabbit IgG (AS014, ABclonal), HRP-conjugated goat anti-mouse IgG (AS003, ABclonal), HRP-conjugated goat anti-mouse IgG light chain (AS062, ABclonal), 488-conjugated goat anti-rabbit IgG (AS053, ABclonal), 594-conjugated goat anti-mouse IgG (AS054, ABclonal).

Full-length viral coding regions of SARS-CoV-2 structural proteins (NCBI RefSeq accession number: NC_045512.2) were cloned and inserted into the pcDNA3.1-3xFlag or pCAGGS-HA vector to establish eukaryotic plasmids expressing Flag- or HA-tagged viral proteins. UBXN7 and its truncated forms were cloned into pCAGGS-HA, and subsequently cloned into pet-28a for prokaryotic expression, or plenti-Fc for eukaryotic expression. For knockdown, shUBXN7 (sequence shown in S1 Table) was inserted into the pLKO.1 vector, and the plasmid was verified by sequencing. Lentivirus packaging plasmids (pMD2.G, psPAX2) and pLenti-Luc were purchased from Addgene. HA-Ub ubiquitin plasmids were originally stored in this laboratory. Cells were transfected with various plasmids using the Hieff Trans Liposomal Transfection Reagent (YEASEN) according to the manufacturer’s protocol. Briefly, cells were seeded onto cell culture dishes and grown to approximately 80% confluence, at which point the medium was replaced with fresh complete medium, and the transfection mixture was prepared at a plasmid-to-liposome ratio of 1:2. After 15 min, the transfection mixture was added to the cell cultures. Cells were harvested 48 h post-transfection for subsequent experiments.

### 4.4 Nuclear and Cytoplasmic fractionation

Cellular fractionation was performed using the Nuclear and Cytoplasmic Protein Extraction Kit (Beyotime Biotechnology, P0027) according to the manufacturer’s protocol. Briefly, harvested cells were washed with PBS and lysed in 500 µL ice-cold Cytoplasmic Extraction Buffer containing protease inhibitors. After vortexing for 15 sec and incubating on ice for 15 min, 25 µL Detergent Buffer was added. The homogenate was centrifuged at 12000 g for 5 min at 4°C. The supernatant (cytoplasmic fraction) was collected, and the pellet was resuspended in 100 μL Nuclear Extraction Buffer with vortexing. After 30 min ice incubation and centrifugation (12000 g, 10 min, 4°C), the supernatant (nuclear fraction) was aliquoted. Protein concentrations were determined by BCA assay.

### 4.5 Immunohistochemical analysis

Formalin-fixed, paraffin-embedded (FFPE) lung tissue sections from 3 healthy donors and 10 SARS-CoV-2-infected patients (approved by the Ethics Committee of Southwest Hospital, Third Military Medical University, Approval No. KY2020298) were subjected to immunohistochemical staining for UBXN7 and SARS-CoV-2 nucleocapsid (N) protein. Briefly, sections were deparaffinized in xylene, rehydrated through graded alcohols, and antigen retrieval was performed by heating in EDTA buffer (for N protein) or 10 mM citrate buffer (pH 6.0, for UBXN7) for 20 min. Endogenous peroxidase activity was quenched with 3% H_2_O_2_ for 15 min. After blocking with 5% BSA for 1 h at 25 °C, sections were incubated overnight at 4 °C with primary antibodies against UBXN7 (1:500) or SARS-CoV-2 N protein (1:10000). Subsequently, HRP-conjugated secondary antibody (1:1000) was applied for 1 h at 37 °C, followed by DAB chromogenic development and hematoxylin counterstaining. Slides were imaged under a light microscope and normalized with nuclear staining intensity by ImageJ software (version 1.54p).

### 4.6 Quantitative real-time PCR analysis (RT-qPCR)

The experimental groups were infected with SARS-CoV-2 VLPs (MOI = 1), HCoV-229E (MOI = 0.5), and HCoV-NL63 (MOI = 0.5). Total RNA was extracted using TRIzol reagent (ThermoFisher) (1x10⁶ cells/mL TRIzol). Chloroform (approximately 200 µL per mL of TRIzol) was added to the lysate, followed by vigorous vortexing. After incubation at 25 °C for 5 min, the mixture was centrifuged (12000 g, 4 °C, 15 min) to achieve phase separation. The aqueous phase containing RNA was carefully transferred to a new tube. An equal volume of isopropanol was added to the aqueous phase, followed by gentle mixing, and incubation for 10 min. The RNA was then pelleted by centrifugation (12000 g, 4 °C, 10 min), washed with 70% ethanol, and dissolved in RNase-free water. cDNA was synthesized using a reverse transcription kit (YEASEN) according to the manufacturer’s instructions. Quantitative polymerase chain reaction (qPCR) was performed using SYBR Green qPCR Master Mix (YEASEN). Gene expression was assessed using a PCR detection system (ROCGENE) according to the reagent instructions; the appropriate number of cycles required for amplifying the target gene was set, and the relative expression of the target gene was calculated via the comparative cycle threshold (2^-ΔΔCT^) method. Primer sequences are listed in S1 Table.

### 4.7 Pseudovirus packaging and pseudovirus entry assay

The packaging of pseudotyped retroviruses bearing coronavirus spikes was performed as previously described [28]. Briefly, pseudoviruses were generated by co-transfecting HEK293T cells with the luciferase-expressing plasmid pLenti-Luc, the backbone plasmid psPAX2, and a plasmid encoding the coronavirus spike protein. Forty-eight hours post-transfection, the cell supernatant was collected, filtered to remove cellular debris, and used as the pseudovirus stock.

For the pseudovirus entry assay, the packaged pseudoviruses were incubated with target cells. After 8 h of incubation at 37 °C, the medium was replaced, and the cells were incubated for a further 48 h. The cells were then washed with PBS and lysed. The cell lysates were transferred to opaque 96-well plates, followed by the addition of luciferase substrate (Promega). Luciferase activity was measured using a microplate reader (TECAN).

### 4.8 Immunofluorescence and confocal immunofluorescence microscopy

After plasmid transfection, cells were seeded onto glass slides and cultured for 24 h until reaching approximately 60% confluence. The cells were then infected with SARS-CoV-2 GFP/∆N VLPs (MOI = 2), HCoV-229E (MOI = 1), or HCoV-NL63 (MOI = 1) at a multiplicity of infection (MOI) of 1. At 24 h post-infection, the cells were washed with PBS and fixed with 4% paraformaldehyde for 15 min. Permeabilization was performed using 0.3% Triton X-100, followed by blocking with 5% goat serum. The cells were incubated with specific primary antibodies at 4 °C overnight, and then with fluorophore-conjugated secondary antibodies at 37 °C for 2 h. Nuclei were stained with DAPI. Images were acquired using a confocal laser scanning microscope (LSCM, Leica SP8).

### 4.9 Immunoprecipitation (IP) and western blotting

To investigate the interaction between exogenous UBXN7 and SARS-CoV-2 N protein, HEK293T cells were cultured in dishes to 70% confluence, and then transfected with specific plasmids according to the experimental design. Total proteins were extracted from the transfected cells using cell lysis buffer containing protease inhibitors. The whole-cell lysates were mixed with specific antibodies (targeting the protein of interest) and Protein A/G agarose beads, and the mixture was incubated at 4 °C overnight to ensure the binding of antibodies to the target proteins and the stable association of the target proteins with the beads. After the reaction, the beads were extensively washed with lysis buffer to remove unbound proteins. Following centrifugation, the supernatant was discarded, and 1 × protein loading buffer (containing SDS and reducing agents) was added. The mixture was heated at 100 °C for 10 min to elute the target proteins bound to the beads.

To investigate the endogenous interaction between UBXN7 and SARS-CoV-2 N protein, HPAEpiC cell were infected with SARS-CoV-2 VLPs at a multiplicity of infection (MOI) of 1 for 24 hours. Cells were lysed in lysis buffer supplemented with protease inhibitor cocktail to preserve native protein complexes. Lysates were incubated with anti-UBXN7 antibody or normal rabbit IgG control overnight at 4°C, followed by precipitation with Protein A/G agarose beads. Immuno-precipitates were washed three times with lysis buffer and eluted. Proteins were separated by SDS-PAGE and transferred to PVDF membranes for immunoblotting using anti-N protein antibody.

The protein samples were loaded into gel wells, with standard protein molecular weight markers used as references. Electrophoresis was performed at 120 V to separate the proteins, followed by transfer from the gel to PVDF membrane at 4 °C for 2 h with a transfer current of 200 mA. The membrane was blocked with 5% skim milk at 25 °C for 1 h. Subsequently, the membrane was incubated with a primary antibody solution specific to the target protein at 4 °C overnight. The next day, the membrane was washed with PBST, incubated with a secondary antibody at 25 °C for 2 h, and washed again with PBST. Finally, the membrane was visualized using an ECL chemiluminescence imaging system.

### 4.10 Liquid chromatography-Mass spectrometry (LC/MS)

Immunoprecipitated proteins for LC-MS analysis were separated by SDS-PAGE and then stained with Coomassie Brilliant Blue for protein visualization. Protein bands were cut from the gel, destained, and then digested with trypsin. The concentration was set at 50 ng/μL, and the incubation was typically carried out for 12 h at 37 °C to ensure complete enzymatic digestion of the proteins into peptides. Dithiothreitol (Sigma Aldrich) solution was added, followed by incubation in a water bath for 1 h. Peptides were dissolved in sample dissolving solution (0.1% formic acid), and each group of samples was detected and analyzed by liquid chromatography (Easy-nLC1200, Thermo Fisher Scientific).

### 4.11 Protein purification and pull down assay

The constructed His-tagged and Fc-tagged eukaryotic expression plasmids were transfected into HEK293F cells. After transfection, the cells were collected by low-temperature centrifugation. The cells were resuspended in lysis buffer and subjected to ultrasonic disruption. Following lysis, the mixture was centrifuged at 10000 g to remove cell debris and insoluble materials, and the supernatant was collected. The supernatant was sequentially purified using a Ni-NTA column and a Superdex200 gel filtration column (GE Healthcare). The purified protein samples were mixed with 1 × protein loading buffer and heated at 100 °C for 10 min to ensure complete denaturation. The samples were then loaded onto an SDS-PAGE gel for electrophoretic separation and analyzed by Coomassie Brilliant Blue staining.

The prokaryotic recombinant SARS-CoV-2 N protein was produced by cloning the full-length N gene (NCBI Reference Sequence: NC_045512.2) into the pET-28a (+) vector with an N-terminal 6 × His tag. The construct was transformed into E. coli BL21 (DE3) cells, and protein expression was induced with 0.2 mM IPTG at 16°C for 16 hours. Cells were lysed by sonication in binding buffer (20 mM Tris-HCl, 500 mM NaCl, 20 mM imidazole, pH 8.0), and the His-tagged N protein was purified using Ni-NTA affinity chromatography.

To form the protein complex, purified UBXN7-his and SARS-CoV-2 N-Fc proteins were co-incubated. The His-tagged UBXN7 protein and Fc-tagged N protein were mixed and incubated overnight at 4 °C with gentle shaking to allow sufficient binding. The UBXN7-his protein was enriched by binding to Ni-conjugated magnetic beads, and the mixture was incubated on a shaker at 4 °C for 1–2 h to ensure thorough binding of the beads to the target protein. After washing, the beads were collected for protein elution. The beads were resuspended in 1 × protein loading buffer and heated to 100 °C for 5 min to ensure complete elution of the target protein from the beads. The eluted samples were collected and analyzed by western blotting.

### 4.12 RNA immunoprecipitation (RIP)

According to the experimental groups, SARS-CoV-2 ΔN trVLPs (MOI = 1) were used to infect either normal HEK293T-N cells, HEK293T-N cells with UBXN7 knockdown, or cells transfected with various N mutant plasmids. After 48 h of culture, cell pellets were collected and lysed using RIP lysis buffer. The N protein was immunoprecipitated using antibodies and agarose beads to form an agarose bead-N-gRNA immunoprecipitation complex. RNA was eluted and purified from the immunoprecipitation complex, and the abundance of gRNA was detected by qPCR using specific primers, following the manufacturer’s instructions (Beyotime, P1801S). The eluted RNA was quantitatively analyzed by qRT-PCR.

### 4.13 Statistical analysis

Independent sample t-test or one-way analysis of variance (ANOVA) was used to analyze the data. In the Figures, significant differences are denoted as * P < 0.05, ** P < 0.01 and *** P < 0.001. In addition, “ns” denotes no significance.

## Supporting information

S1 Fig Construction of SARS-CoV-2 virus-like particles (trVLPs) in trans-cell culture system. (A) The construction flow chart of SARS-CoV-2 GFP/ΔN trVLPs. (B-G) Amplification and purification of trVLPs genomic sequence fragments. (H-J) Viral full-length RNA (FL-RNA) and N gene mRNA produced by in vitro transcription. (K) Fluorescence microscopy analysis of Vero-N cells infected with SARS-CoV-2 GFP/ΔN. (L) Quantification of mRNA levels in Vero cells transfected with shUBXN7 by qPCR. (M) Evaluation of UBXN7 knockdown efficiency in Vero cells by western blot. Protein quantification was performed using Image J software with normalization.
(TIF)

S2 Fig UBXN7 does not affect the replication of VSV and RSV. (A-B) HEK293T and Calu-3 cells were infected with VSV-GFP for 24h and 48h, and the number of infected cells and fluorescence intensity were observed under a fluorescence microscope. (C-D) A549 and HEP-2 cells were infected with RSV-mcherry for 24 h and 48 h, and the number of infected cells and fluorescence intensity were observed under fluorescence microscopy.
(TIF)

S3 Fig UBXN7 interacts with nucleocapsid proteins of human coronaviruses 229E and NL63 in HEK293T cells. (A) HEK293T cells were co-transfected with plasmids encoding HA-UBXN7 and Flag-229E N protein. Cell lysates were subjected to immunoprecipitation using anti-HA antibody, followed by western blot with anti-HA and anti-Flag antibodies. (B) Interaction analysis between HA-UBXN7 and Flag-NL63 N protein performed similarly as in (A). (C) Affinity chromatography purification peak diagram and Coomassie brilliant blue stained gel image of UBXN7 protein. (D) GO pathway analysis of LC-MS data.
(TIF)

S4 Fig UBXN7 does not affect the expression levels of IFN-α and IFN-β. (A-B) Transfect UBXN7 plasmid for 24 hours, collect SARS-CoV-2 trVLP infection samples at different time points, and measure the mRNA levels of IFN-α and IFN-β. (C-F) Transfect UBXN7 plasmid for 24 hours, collect HCoV-229E infection samples at different time points, and measure the mRNA levels of IFN-α and IFN-β in different cells. (G-J) Transfect UBXN7 plasmid for 24 hours, collect HCoV-NL63 infection samples at different time points, and measure the mRNA levels of IFN-α and IFN-β in different cells. The significant differences are * P < 0.05, ** P < 0.01 and *** P < 0.001. In addition, “ns” means no significance.
(TIF)

S1 Table qRT-PCR primers used in this study. (DOCX)
