# Supplementary figures and images for: UBXN7 facilitates SARS-CoV-2 replication via inhibiting the K48-linked ubiquitination of viral N protein
Source: PLoS Pathog. 2025 Oct 14;21(10):e1013593. doi: 10.1371/journal.ppat.1013593 (PMC12520364; doi:10.1371/journal.ppat.1013593)

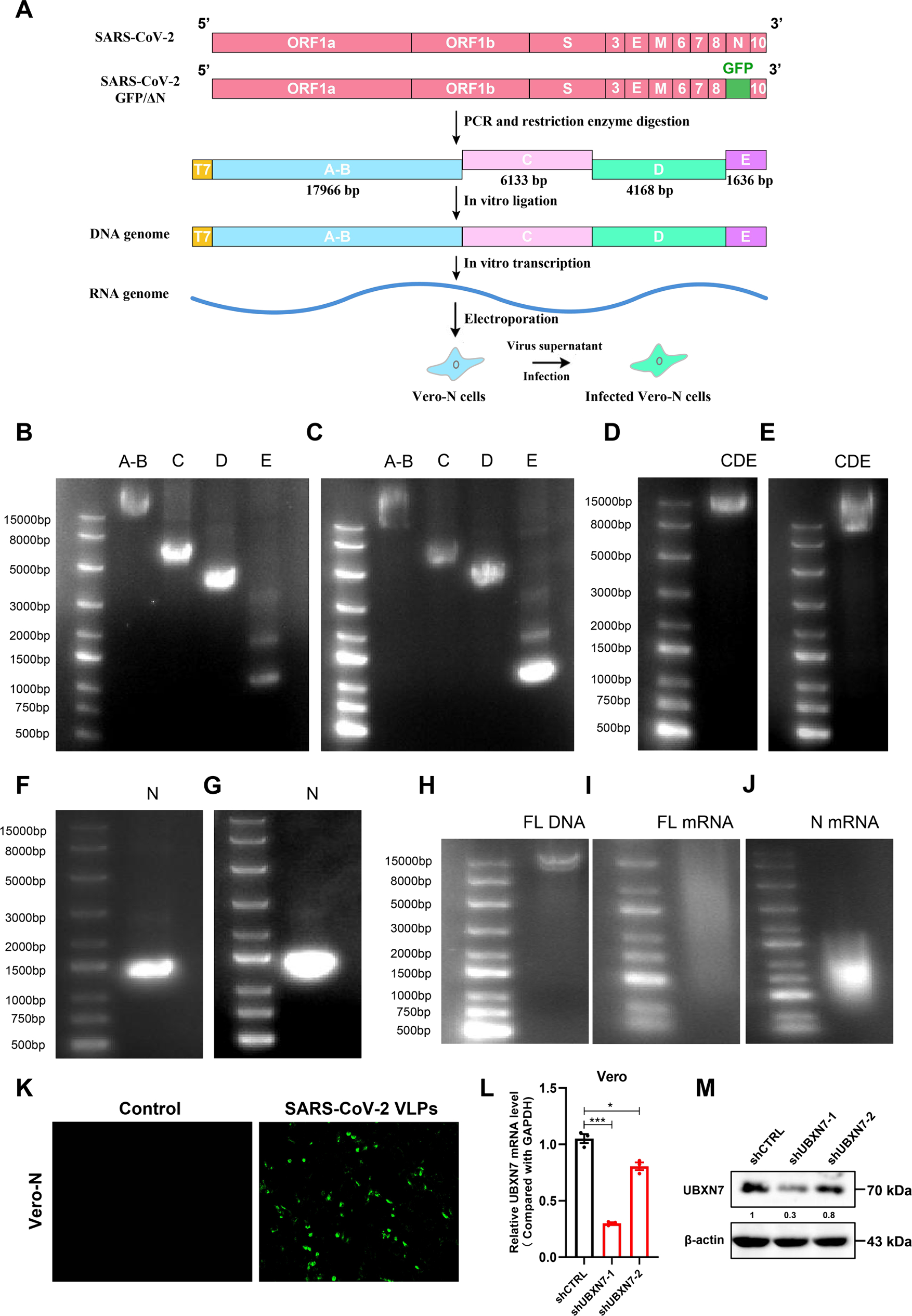

Supplement: S1 Fig — (A) The construction flow chart of SARS-CoV-2 GFP/ΔN trVLPs. (B-G) Amplification and purification of trVLPs genomic sequence fragments. (H-J) Viral full-length RNA (FL-RNA) and N gene mRNA produced by in vitro transcription. (K) Fluorescence microscopy analysis of Vero-N cells infected with SARS-CoV-2 GFP/ΔN. (L) Quantification of mRNA levels in Vero cells transfected with shUBXN7 by qPCR. (M) Evaluation of UBXN7 knockdown efficiency in Vero cells by western blot. Protein quantification was performed using Image J software with normalization. (TIF) [file ppat.1013593.s001.tif]

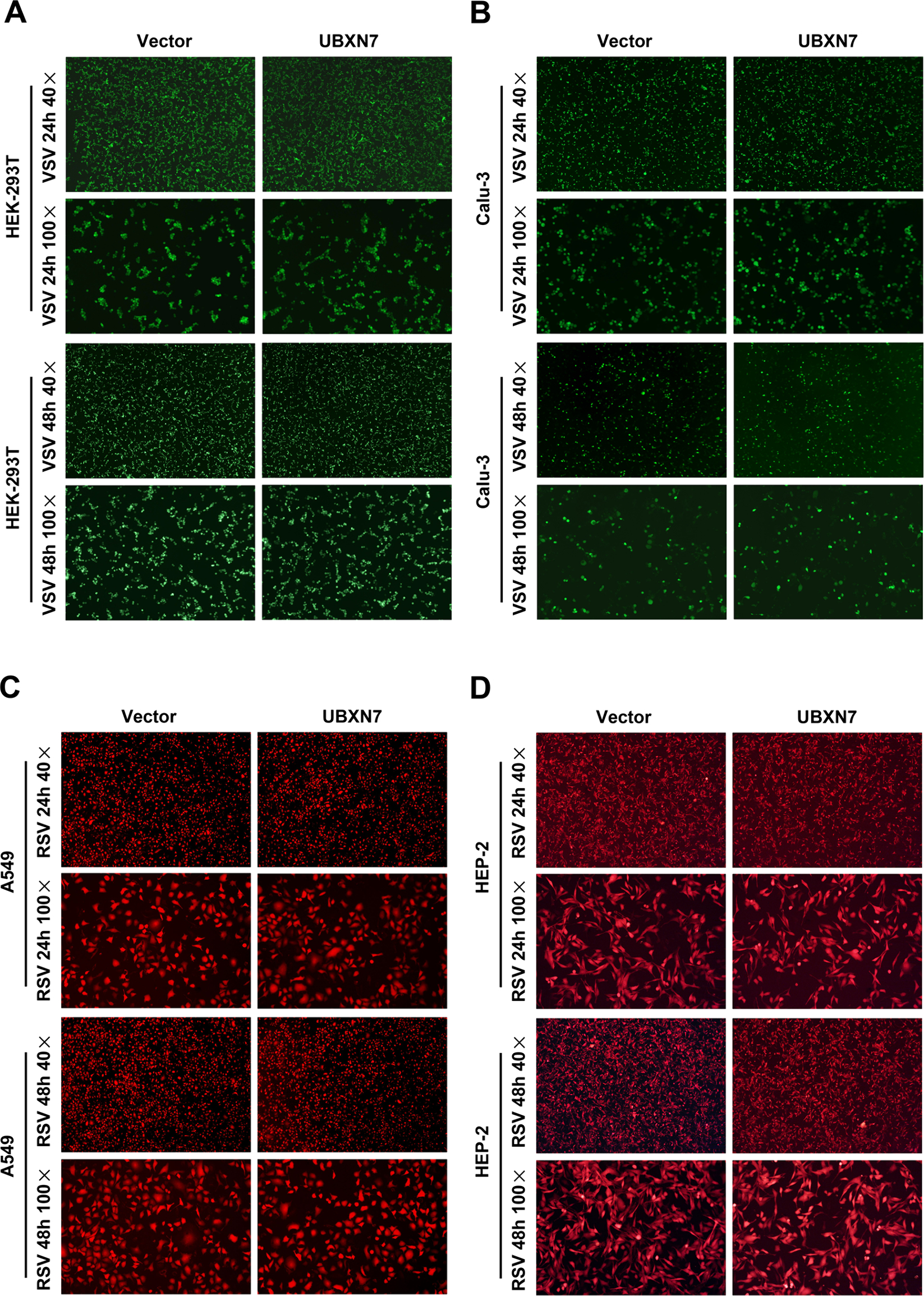

Supplement: S2 Fig — (A-B) HEK293T and Calu-3 cells were infected with VSV-GFP for 24h and 48h, and the number of infected cells and fluorescence intensity were observed under a fluorescence microscope. (C-D) A549 and HEP-2 cells were infected with RSV-mcherry for 24 h and 48 h, and the number of infected cells and fluorescence intensity were observed under fluorescence microscopy. (TIF) [file ppat.1013593.s002.tif]

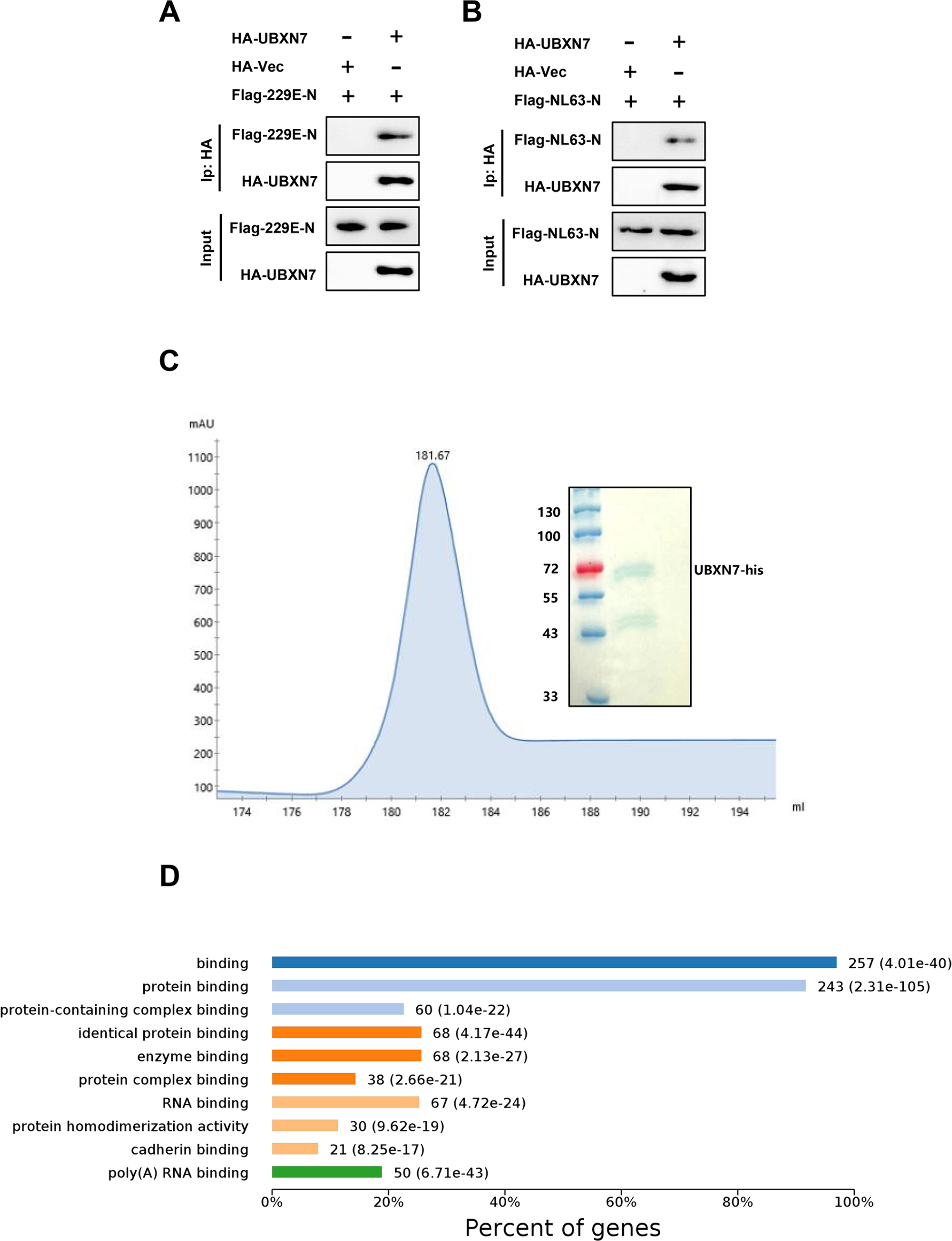

Supplement: S3 Fig — (A) HEK293T cells were co-transfected with plasmids encoding HA-UBXN7 and Flag-229E N protein. Cell lysates were subjected to immunoprecipitation using anti-HA antibody, followed by western blot with anti-HA and anti-Flag antibodies. (B) Interaction analysis between HA-UBXN7 and Flag-NL63 N protein performed similarly as in (A). (C) Affinity chromatography purification peak diagram and Coomassie brilliant blue stained gel image of UBXN7 protein. (D) GO pathway analysis of LC-MS data. (TIF) [file ppat.1013593.s003.tif]

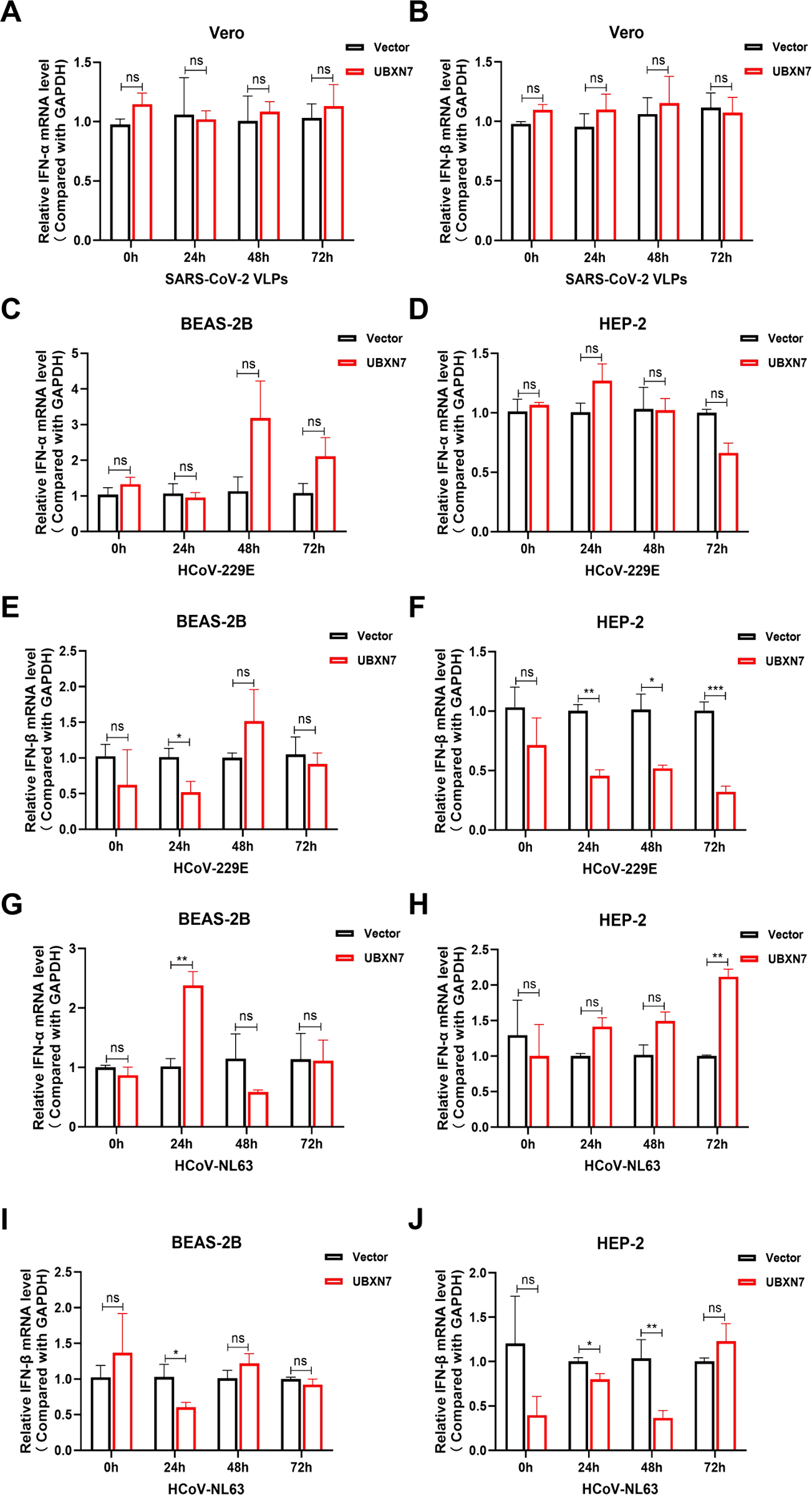

Supplement: S4 Fig — (A-B) Transfect UBXN7 plasmid for 24 hours, collect SARS-CoV-2 trVLP infection samples at different time points, and measure the mRNA levels of IFN-α and IFN-β. (C-F) Transfect UBXN7 plasmid for 24 hours, collect HCoV-229E infection samples at different time points, and measure the mRNA levels of IFN-α and IFN-β in different cells. (G-J) Transfect UBXN7 plasmid for 24 hours, collect HCoV-NL63 infection samples at different time points, and measure the mRNA levels of IFN-α and IFN-β in different cells. The significant differences are * P < 0.05, ** P < 0.01 and *** P < 0.001. In addition, “ns” means no significance. (TIF) [file ppat.1013593.s004.tif]
