# Supplementary material for: UBXN7 facilitates SARS-CoV-2 replication via inhibiting the K48-linked ubiquitination of viral N protein
Source: PLoS Pathog. 2025 Oct 14;21(10):e1013593. doi: 10.1371/journal.ppat.1013593 (PMC12520364; doi:10.1371/journal.ppat.1013593)
Supplement: S1 Table — (DOCX) [file ppat.1013593.s005.docx]

**Supplementary Table 1 Primer sequence information**

| **Primer name** | **Sequence** | |
| --- | --- | --- |
| sh-CTRL | | CAACAAGATGAAGAGCACCAA |
| sh-UBXN7 | | GGTGGAACCAGAACCATTATT |
| sh-UBXN7-1 | | GACGGCCTGCACGTTCAATTT |
| qpcr-229E-F | | CGCAAGAATTCAGAACCAGAG |
| qpcr-229E-R | | GGGAGTCAGGTTCTTCAACAA |
| qpcr-NL63-F | | AGGACCTTAAATTCAGACAACGTTCT |
| qpcr-NL63-R | | GATTACGTTTGCGATTACCAAGACT |
| qpcr-UBXN7-F | | AGCCACAAACCACCAAGGAT |
| qpcr-UBXN7-R | | GCCTGCCTCTTGCAATGTAAT |
| qpcr-SARS-CoV-2-F | | CGAAAGGTAAGATGGAGAGCC |
| qpcr-SARS-CoV-2-R | | TGTTGACGTGCCTCTGATAAG |
| qpcr-IFN-α-F | | TCCAGAAGGCTCCAGCCATCT |
| qpcr-IFN-α-R | | TGCATCACACAGGCTTCCAAG |
| qpcr-IFN-β-F | | CCAACAAGTGTCTCCTCCAA |
| qpcr-IFN-β-R | | ATAGTCTCATTCCAGCCAGT |
| qpcr-GAPDH-F | | GAAGGTGAAGGTCGGAGTCA |
| qpcr-GAPDH-R | | TTGAGGTCAATGAAGGGGTC |
